# Involvement of *17β-hydroxysteroid dehydrogenase type* gene *1* 937 A>G polymorphism in infertility in Polish Caucasian women with endometriosis

**DOI:** 10.1007/s10815-017-0911-9

**Published:** 2017-04-12

**Authors:** Maciej Osiński, Adrianna Mostowska, Przemyslaw Wirstlein, Jana Skrzypczak, Paweł Piotr Jagodziński, Malgorzata Szczepańska

**Affiliations:** 1 Department of Obstetrics, Gynecology and Gynecological Oncology, Division of Reproduction, Poznan University of Medical Sciences, 33 Polna St, 60-535 Poznań, Poland; 2 Department of Biochemistry and Molecular Biology, Poznan University of Medical Sciences, Poznan, Poland

**Keywords:** Endometriosis, *HSD17B1*, Polymorphism

## Abstract

**Purpose:**

Endometriosis is considered to be an estrogen-related chronic inflammatory disease. The 17β-hydroxysteroid dehydrogenase 1 (HSD17B1) converts estrone to 17β estradiol. The role of *HSD17B1* 937 A>G (rs605059) single nucleotide polymorphism (SNP) in development of endometriosis is still disputable. This study evaluated the association of the *HSD17B1* 937 A>G (rs605059) SNP with infertile women affected by endometriosis from Polish Caucasian population.

**Methods:**

The genotyping of cases (*n* = 290) and fertile women (*n* = 410) was conducted by high-resolution melting curve analysis.

**Results:**

Statistical analysis demonstrated that the *HSD17B1* 937 A>G SNP is associated with endometriosis in stages I and II. The *p*_trend_ and *p*_allelic_ values calculated for the *HSD17B1* 937 A>G polymorphism were statistically significant and were equal to 0.001 and 0.0009, respectively. There was a significant association for the dominant model: (AG + GG vs AA) OR = 1.973 (95% CI = 1.178–3.304), *p* = 0.009, and for the recessive model: (GG vs AG + AA) OR = 1.806 (95% CI = 1.178–2.770), *p* = 0.006*.* However, we did not find statistical association of *HSD17B1* 937 A>G polymorphism with all infertile women with endometriosis or infertile women with endometriosis in stages III and IV.

**Conclusion:**

Our genetic study demonstrated *HSD17B1* 937 G variant as a risk factor for infertility in women with stage I and II endometriosis in Polish Caucasian patients.

**Electronic supplementary material:**

The online version of this article (doi:10.1007/s10815-017-0911-9) contains supplementary material, which is available to authorized users.

## Introduction

Endometriosis is a benign disease affecting women of childbearing age, which is characterized by the presence of ectopic endometrial implants outside the uterine cavity [1]. These implants are found in the pelvis, uterosacral ligaments, ovaries, rectovaginal septum, and pouch of Douglas. This disorder affects 5–10% of women in reproductive age, with incidence of 30–50% in infertile women [2–4]. The exact pathogenesis of endometriosis is still unclear. Retrograde menstruation is recognized as a possible mechanism in the development of this disease. The retrograde menstruation is observed in 90% of women of childbearing age; however, merely 10% of women appeared to develop endometriosis [5].

This suggests that several factors are involved in the pathogenesis of endometriosis, which increase persistence of the endometriotic lesions. Both the occurrence and progression of endometriosis are related to abnormal expression of proteins involved in angiogenesis, cell growth, immune response, reduced progestin, and increased estrogenic activity [6].

Endometriosis is considered as an estrogen-related chronic inflammatory disease [4]. There is increased evidence demonstrating that endometriotic implants contain aromatase, which converts androgen to estrogen [4, 6–8].

Recent studies have demonstrated the presence of 17β-hydroxysteroid dehydrogenase (HSD17Bs) enzyme family expression in endometriotic lesions. HSD17Bs are involved in either the oxidation or reduction of sex steroids [9]. HSD17B1 carries out the conversion of estrone (E1) to 17β estradiol (E2), which is a more biologically active estrogen [9].

It has been suggested that genetic variants of *HSD17B1*, specifically the 937 A>G (rs605059) single nucleotide polymorphism (SNP), may contribute to endometriosis [10, 11]. This transition is located in exon 6 and alters serine to glycine at position 312 [12, 13]. Studies conducted to this day inconsistently demonstrated *HSD17B1* 937 A>G SNP as a genetic risk factor of endometriosis [10, 11, 14, 15]. Therefore, our study aimed to evaluate the contribution of *HSD17B1* 937 A>G SNP to infertility in women with endometriosis in the Polish population.

## Materials and methods

### Study population

Peripheral blood samples were obtained from infertile women with endometriosis and control women of similar age from the Gynecologic and Obstetrical University Hospital, Division of Reproduction at Poznan University of Medical Sciences, Poland. A case-control study design was used in 290 patients with endometriosis and 410 matched controls (Table 1). The patients with infertility and endometriosis underwent laparoscopy and had a histologically confirmed diagnosis at the Gynecologic and Obstetrical University Hospital, Division of Reproduction at Poznan University of Medical Sciences, Poland. Patients with endometriosis were divided into two subgroups according to the revised American Society for Reproductive Medicine (rASRM) classification system [16]; *n* = 126 patients (43.45%) had minimal or mild endometriosis (stages I–II) and *n* = 157 (54.14%) had moderate or severe endometriosis (stages III–IV); *n* = 7 (2.41%) patients had undefined stage of endometriosis (Table 1). The control group was encompassed of healthy women (*n* = 410), without history of infertility, who had a cesarean section performed (Table 1).

**Table 1:** Characteristics of the populations of infertile women with endometriosis and fertile healthy women

Characteristics	Infertile women with endometriosis	Fertile healthy women
Numbers	290	410
Age (years)	33 (21–37)^a^	32 (18–37)^a^
Parity	NA	1 (1–5)^a^
Duration of infertility (years)	3 (1–8)^a^	NA
rASRM (stage)^b^	Stages I and II (*n* = 126) Stages III and IV (*n* = 157) Undefined (*n* = 7)	NA

*NA* not applicable

^a^Median (range)

^b^Revised American Society for Reproductive Medicine (rASRM) [16]

The inclusion and exclusion criteria for the infertile women with endometriosis and the women without the disease were previously described [17]. Inclusion criteria for infertile women with diagnosed endometriosis were regular menses, no anatomical changes in the reproductive tract, no hormonal treatments, and a minimum of 1 year of infertility with a current desire for conception. Exclusion criteria were mechanical distortion of the endometrial cavity by fibroids, bilateral tubal occlusion, male factor infertility, adenomyosis, polycystic ovary syndrome (PCOS), and benign or malignant gynecological diseases. All included patients with endometriosis had laparoscopic and histological diagnosis of endometriosis. Inclusion criteria for fertile control women were cesarean section performed, regular menses, no anatomical changes in the reproductive tract, no hormonal treatments, and at least one child born no more than 1 year before study (Table 1). Exclusion criteria were signs of past or present inflammation, pelvic abnormalities, endometriosis, adenomyosis, PCOS, or any other benign or malignant gynecological diseases, which was confirmed during surgical exploration. Both patients with endometriosis and healthy controls were all Caucasians of Polish ancestry (Table 1).Written informed consent was obtained from all participating individuals. The study was conducted in accordance with the code of ethics of the Declaration of Helsinki and obtained the approval of the Local Ethical Committee of Poznan University of Medical Sciences.

### Genotyping

Genomic DNA was isolated from peripheral blood leukocytes by salt extraction. Genotyping was conducted by high-resolution melting (HRM) curve analysis on the LightCycler 480 system (Roche Diagnostics, Mannheim, Germany). Genomic DNA was amplified with the use of specific primers F: CCTGGGGCAGAGGACGAG and R: AAGAAGGGCGCGGGAGAC. The annealing temperature was 66 °C and the PCR product size was 113 bp. Amplified DNA fragments were then subjected to HRM with 0.1 °C increments in temperatures ranging from 85 to 98 °C. The genotyping quality was evaluated by repeated genotyping of 10% randomly selected samples.

### Data analysis

Hardy-Weinberg equilibrium (HWE) was assessed by Pearson’s goodness-of-fit chi-square (*χ* ^2^) statistic. The SNP was studied for associations with endometriosis using the Cochran-Armitage trend test. Differences in the allele and genotype frequencies between the cases and controls were calculated using *χ* ^2^ analysis. The odds ratio (OR) and associated 95% confidence intervals (95% CI) were also computed. The *p* values <0.05 were considered as statistically significant. Power calculations were evaluated using Quanto software (Gauderman WJ, Morrison JM. QUANTO 1.2: a computer program for power and sample size calculations for genetic-epidemiology studies, URL http://biostats.usc.edu/software).

## Results

### The comparison of *HSD17B1* 937 A>G genotype and allele frequencies between all infertile women with endometriosis in stages I, II, III, and IV and fertile healthy women

The distribution of *HSD17B1* 937 A>G genotypes did not differ from HWE between patients (*p* = 0.378) and controls (*p* = 0.665) for all women with endometriosis. The prevalence of the genotype and allele frequencies, OR, and 95% CI computed for the *HSD17B1* 937 A>G for both fertile healthy women and all women with endometriosis stages I, II, III, and IV are stated in Table 2. Statistical analysis demonstrated that the *HSD17B1* 937 A>G polymorphism is not associated with all infertile women with endometriosis. The *p*_trend_ and *p*_allelic_ values computed for *HSD17B1* 937 A>G SNP were not statistically significant and were equal to 0.195 and 0.181, respectively. The OR for dominant model: (AG + GG vs AA) was 1.152 (95% CI = 0.819–1.619), *p* = 0.416, and OR for recessive model: (GG vs AG + AA) was 1.260 (95% CI = 0.896–1.771), *p* = 0.184.

**Table 2:** *HSD17β1* 937 A>G (s605059) polymorphism as the risk of all stages cumulatively, stages I and II, and stages III and IV of endometriosis when compared with fertile healthy women

	Cases						Controls									
Stage of endometriosis	Genotypes			Alleles^a^			Genotypes			Alleles^a^			*p*_trend_ value	*p*_allelic_ value	OR_AG+GGvsAA_ (95% CI)^b^; *p* value^d^	OR_GGvsAG+AA_ (95% CI)^c^; *p* value^d^
		No.	Freq.		No.	Freq.		No.	Freq.		No.	Freq.				
All stages *n* = 290	AA	74	0.25	A	281	0.48	AA	116	0.28	A	427	0.52	0.195	0.181	1.152 (0.819–1.619); 0.416	1.260 (0.896–1.771); 0.184
	AG	133	0.46	G	299	0.52	AG	195	0.48	G	393	0.48				
	GG	83	0.29				GG	99	0.24							
Stages I and II *n* = 126	AA	21	0.17	A	101	0.40	AA	116	0.28	A	427	0.52	*0.001*	*0.0009*	*1.973* (*1.178*–*3.304*); *0.009*	*1.806* (*1.178*–*2.770*); *0.006*
	AG	59	0.47	G	151	0.60	AG	195	0.48	G	393	0.48				
	GG	46	0.36				GG	99	0.24							
Stages III and IV *n* = 157	AA	51	0.32	A	174	0.55	AA	116	0.28	A	427	0.52	0.326	0.313	0.820 (0.551–1.220); 0.327	0.868 (0.558–1.351); 0.531
	AG	72	0.46	G	140	0.45	AG	195	0.48	G	393	0.48				
	GG	34	0.22				GG	99	0.24							

For seven cases, the stage of endometriosis is unknown. Significant results are in italics

^a^Underline denotes the minor allele (MA)

^b^Dominant model (G is the risk allele)

^c^Recessive model (G is the risk allele)

^d^Chi-square analysis

### The comparison of *HSD17B1* 937 A>G genotype and allele frequencies between infertile women with endometriosis in stages I and II and fertile healthy women

The prevalence of the genotype and allele frequencies, OR, and 95% CI computed for the *HSD17B1* 937 A>G for both fertile healthy women and women with endometriosis stages I and II are stated in Table 2. The *p*_trend_ and *p*_allelic_ values calculated for the *HSD17B1* 937 A>G polymorphism were statistically significant and were equal to 0.001 and 0.0009, respectively. We observed significant association for the dominant model: (AG + GG vs AA) OR = 1.973 (95% CI = 1.178–3.304), *p* = 0.009, and for recessive model: (GG vs AG + AA) OR = 1.806 (95% CI = 1.178–2.770), *p* = 0.006. A power analysis predicted sufficient power to detect an association of the *HSD17B1* 937 A>G polymorphism with a genetic effect of 1.9 or more for the recessive and a genetic effect of 2.1 or more for the dominant model in infertile women with endometriosis in stages I and II (Supplementary file 1).

### The comparison of *HSD17B1* 937 A>G genotype and allele frequencies between infertile women with endometriosis in stages III and IV and fertile healthy women

The prevalence of the genotype and allele frequencies, OR, and 95% CI computed for the *HSD17B1* 937 A>G for both fertile healthy women and women with endometriosis stages III and IV are stated in Table 2. There was no observed contribution of *HSD17B1* 937 A>G to endometriosis to stages III and IV, and the *p*_trend_ and *p*_allelic_ values were not statistically significant and were equal to 0.326 and 0.313, respectively (Table 2). The OR for dominant model: (AG + GG vs AA) was OR = 0.820 (95% CI = 0.551–1.220), *p* = 0.327, and for recessive model: (GG vs AG + AA) was OR = 0.868 (95% CI = 0.558–1.351), *p* = 0.531.

## Discussion

The physiological role of E2 in the menstrual cycle has already been well determined [18]. Estrogen is the main steroid of the proliferative phase of the reproductive cycle [18]. E2 initiates a significant proliferation of the endometrial tissue and supports the growth of the endometrial glands before ovulation, preparing endometrium for the action of progesterone [18]. However, abnormal production of estrogen contributes to various estrogen-related diseases including endometriosis [19, 20]. The significant function of E2 in the development of this disease has been well documented in animal models including rodent and baboon models [21, 22]. More endometriotic lesions were developed in E2-treated mice than control animals [21]. Recently, Nair et al. employed antiprogestin treatment which resulted in unopposed estrogenicity and development of spontaneous endometriosis in baboons [22].

There are many human studies that demonstrate upregulation of local estrogen production in endometriotic tissue, mainly by increased catalytic aromatase activity [6, 7, 23]. Moreover, the endometriotic implants have increased E2 levels as compared to E1 throughout the menstrual cycle [24]. The treatments of endometriosis include the reduction of estrogens to decrease their stimulatory effect on the endometrium. These treatments include progestins, oral contraceptives, and antagonists of gonadotropin-releasing hormone [25, 26].

The expression of different *HSD17B*s including *HSD17B1* responsible for estrogen metabolism was well documented in eutopic and ectopic endometriotic tissue [27–29]. Inhibitors of HSD17B1 are considered in treatment of endometriosis [27]. In our study, we found significant association of *HSD17B1* 937 G variant with infertile women having endometriosis in stages I and II, but not in all of the infertile women with endometriosis or infertile women with endometriosis in stages III and IV.

Our results are contradictory to Lamp et al. (2010) who found *HSD17B1* A variant as a risk of endometriosis at stage I–II disease, in Estonian population [10]. Our findings are also inconsistent with Tsuchiya et al. (2005) who demonstrated contribution of A allele of *HSD17B1* to endometriosis at stages III and IV [11]. There are studies which did not find association of *HSD17B1* 937 A>G polymorphism with endometriosis [15, 16]. The study conducted in Taiwanese Han and Brazilian population also did not indicate *HSD17B1* 937 A>G SNP contribution to endometriosis [15, 30]. Trabert et al. (2011) did not observe contribution of *HSD17B1* rs2676530 and rs676387 polymorphisms to endometriosis patients from western Washington cohort, including Caucasian, African, and Asian American individuals [14]. A recent meta-analysis confirms lack of *HSD17B1* rs605059 polymorphism association with endometriosis in the overall population as well as in subgroup ethnicities [31]. The different effect of *HSD17B1* 937 A>G on the development of endometriosis in distinct ethnicities may have also resulted from genetic heterogeneity, size of the studied groups, and different populations’ exposure to environmental factors.

The differences between Estonian [10], Japanese [11], and our *HSD17B1* 937 gene variant association might be due to linkage disequilibrium of rs605059 with an unknown SNP in Polish Caucasian population. Moreover, our study was conducted in selected group of infertile women with endometriosis at stages I and II in which *HSD17B1* 937 G variant can be a risk of infertility in our evaluated cohort. Recently, Ntostis et al. (2015) has suggested *HSD17B1* 937 G variant being a risk of recurrent spontaneous abortions [32].

The disagreement in role of *HSD17B1* 937 A>G SNP in the development of other diseases has been demonstrated in different populations. They include development of the risk of endometrial and breast cancer [31, 33–35]. The meta-analysis did not show significant association between HSD17B1 rs605059 gene polymorphisms and risks of endometrial cancer [31]. Additionally, the recent meta-analysis conducted by Shi et al. (2016) suggested that the *HSD17B1* 937 G allele may protect from breast cancer development in Caucasians, but not among Asians [35].

It has been demonstrated that women with severe stages of the endometriosis displayed poor ovarian reserve, low oocyte and embryo quality, poor implantation, pelvic anatomy distortion, and mechanical disruptions such as pelvic adhesions [36–38]. These abnormalities account for infertility, which is more likely to be found in women in severe stages of the endometriosis [39]. However, the possible mechanisms by which minimal/mild endometriosis impacts fertility is still elusive [40]. The overproduction of E2 has to be considered as a causative factor of infertility in endometriosis [4, 6–8]. *HSD17B1* 937 A>G SNP might be linked to changes in conversion of E1 to E2, which might account for infertility in women with minimal/mild endometriosis.

Endometriosis is a multifactorial disease that is influenced by multiple genes and a variety of environmental factors, including lifestyle [6]. It has been suggested that moderate and severe endometrioses have greater genetic burden than minimal or mild disease [41]. This may partially explain why *HSD17B1* 937 A>G SNP is associated with minimal/mild but not moderate/severe endometriosis, which in turn will probably require a greater contribution of additional genetic and/or environmental factors for its etiology [41].

To date, the genetic risk factors for endometriosis-related infertility have also included the *ESR1*, *ESR2*, and *luteinizing hormone beta*-*subunit FOXP*, *complement component 3*, *lysyl oxidase-like protein 4*, and *FCRL3* genes [10, 42–46]. Recently, it has been suggested that *CYP17*, *VDR*, *MUC17*, *COX-2*, *WNT4*, *E-cadherin*, *CYP19*, *CYP17*, *TYK2*, *NFKB1*, and MUC2 gene variants also contributed to endometriosis-related infertility [17, 47–55].

## Conclusions

Our study demonstrated the *HSD17B1* 937 G variant as risk factor for infertility in women with endometriosis at stages I and II. Our genetic study was carried out on a relatively small group of infertile women with endometriosis; therefore, the role of this polymorphism should be further studied in women with idiopathic infertility and a larger and independent cohort of infertile women with endometriosis.

### Acknowledgements

This study was supported by grant nos. 502-01-01124182-07474 and 502-14-01110142-41198, Poznan University of Medical Sciences.

## Electronic supplementary material

Supplementary Table 1 (DOCX 20 kb)
