# Supplementary material for: Involvement of 17β-hydroxysteroid dehydrogenase type gene 1 937 A>G polymorphism in infertility in Polish Caucasian women with endometriosis
Source: J Assist Reprod Genet. 2017 Apr 12;34(6):789–94. doi: 10.1007/s10815-017-0911-9 (PMC5445048; doi:10.1007/s10815-017-0911-9)
Supplement: Supplementary file 1 — (DOCX 20 kb) [file 10815_2017_911_MOESM1_ESM.docx]

**Supplementary Table 1. Power analysis**

| **Design:** | unmatched case-control (1:1.41) | | |  |  |
| --- | --- | --- | --- | --- | --- |
| **Sample size:** | 290 cases (endometriosis all types) | | | |  |
| **Population risk:** | 0.04 |  |  |  |  |
| **MAF:** | 0.48 |  |  |  |  |
|  | **Genetic effect** | **Recessive model** | **Dominant model** |  |  |
|  | 0.5 | 0.9276 | 0.9891 |  |  |
|  | 0.7 | 0.4598 | 0.5704 |  |  |
|  | 0.9 | 0.0879 | 0.0946 |  |  |
|  | 1.1 | 0.0827 | 0.0847 |  |  |
|  | 1.3 | 0.3180 | 0.3125 |  |  |
|  | 1.5 | 0.6451 | 0.6058 |  |  |
|  | 1.7 | 0.8706 | 0.8170 |  |  |
|  | 1.9 | 0.9651 | 0.9260 |  |  |
|  | 2.1 | 0.9926 | 0.9723 |  |  |
|  | 2.3 | 0.9987 | 0.9900 |  |  |
|  | 2.5 | 0.9998 | 0.9964 |  |  |
|  | 2.7 | 0.9999 | 0.9987 |  |  |
|  | 2.9 | 0.9999 | 0.9995 |  |  |
|  |  | | |  |  |
| **Design:** | unmatched case-control (1:3.25) | | |  |  |
| **Sample size:** | 126 cases (endometriosis stage I and II) | | | |  |
| **Population risk:** | 0.04 |  |  |  |  |
| **MAF:** | 0.48 |  |  |  |  |
|  | **Genetic effect** | **Recessive model** | **Dominant model** |  |  |
|  | 0.5 | 0.7207 | 0.8977 |  |  |
|  | 0.7 | 0.2855 | 0.3662 |  |  |
|  | 0.9 | 0.0713 | 0.0751 |  |  |
|  | 1.1 | 0.0685 | 0.0695 |  |  |
|  | 1.3 | 0.2027 | 0.1970 |  |  |
|  | 1.5 | 0.4239 | 0.3857 |  |  |
|  | 1.7 | 0.6501 | 0.5718 |  |  |
|  | 1.9 | 0.8182 | 0.7196 |  |  |
|  | 2.1 | 0.9170 | 0.8230 |  |  |
|  | 2.3 | 0.9658 | 0.8903 |  |  |
|  | 2.5 | 0.9870 | 0.9324 |  |  |
|  | 2.7 | 0.9953 | 0.9582 |  |  |
|  | 2.9 | 0.9984 | 0.9739 |  |  |
|  |  | | |  |  |
| **Design:** | unmatched case-control (1:2.61) | | |  |  |
| **Sample size:** | 157 cases (endometriosis stage III and IV) | | | | |
| **Population risk:** | 0.04 |  |  |  |  |
| **MAF:** | 0.48 |  |  |  |  |
|  | **Genetic effect** | **Recessive model** | **Dominant model** |  |  |
|  | 0.5 | 0.7909 | 0.9379 |  |  |
|  | 0.7 | 0.3278 | 0.4185 |  |  |
|  | 0.9 | 0.0752 | 0.0797 |  |  |
|  | 1.1 | 0.0718 | 0.0730 |  |  |
|  | 1.3 | 0.2301 | 0.2241 |  |  |
|  | 1.5 | 0.4826 | 0.4421 |  |  |
|  | 1.7 | 0.7194 | 0.6435 |  |  |
|  | 1.9 | 0.8743 | 0.7895 |  |  |
|  | 2.1 | 0.9518 | 0.8813 |  |  |
|  | 2.3 | 0.9836 | 0.9346 |  |  |
|  | 2.5 | 0.9949 | 0.9642 |  |  |
|  | 2.7 | 0.9985 | 0.9803 |  |  |
|  | 2.9 | 0.9996 | 0.9890 |  |  |
